# Assessing actigraphy performance for daytime sleep detection following stroke: insights from inpatient monitoring in a rehabilitation hospital

**DOI:** 10.1093/sleepadvances/zpae057

**Published:** 2024-07-31

**Authors:** Jiayi E Wang, Jacob Sindorf, Pin-Wei Chen, Jessica Wu, Adrian Gonzales, Megan K O’Brien, Aashna Sunderrajan, Kristen L Knutson, Phyllis C Zee, Lisa Wolfe, Vineet M Arora, Arun Jayaraman

**Affiliations:** School of Medicine, University of Chicago, Chicago, IL, USA; Max Nader Center for Rehabilitation Technologies and Outcomes Research, Shirley Ryan AbilityLab, Chicago, IL, USA; Max Nader Center for Rehabilitation Technologies and Outcomes Research, Shirley Ryan AbilityLab, Chicago, IL, USA; School of Medicine, University of Chicago, Chicago, IL, USA; School of Medicine, University of Chicago, Chicago, IL, USA; Max Nader Center for Rehabilitation Technologies and Outcomes Research, Shirley Ryan AbilityLab, Chicago, IL, USA; Feinberg School of Medicine, Northwestern University, Chicago, IL, USA; School of Medicine, University of Chicago, Chicago, IL, USA; Feinberg School of Medicine, Northwestern University, Chicago, IL, USA; Feinberg School of Medicine, Northwestern University, Chicago, IL, USA; Feinberg School of Medicine, Northwestern University, Chicago, IL, USA; School of Medicine, University of Chicago, Chicago, IL, USA; Max Nader Center for Rehabilitation Technologies and Outcomes Research, Shirley Ryan AbilityLab, Chicago, IL, USA; Feinberg School of Medicine, Northwestern University, Chicago, IL, USA

**Keywords:** stroke, actigraphy, napping, Inpatient Rehabilitation Hospital

## Abstract

**Study Objectives:**

Stroke can result in or exacerbate various sleep disorders. The presence of behaviors such as daytime sleepiness poststroke can indicate underlying sleep disorders which can significantly impact functional recovery and thus require prompt detection and monitoring for improved care. Actigraphy, a quantitative measurement technology, has been primarily validated for nighttime sleep in healthy adults; however, its validity for daytime sleep monitoring is currently unknown. Therefore this study aims to identify the best-performing actigraphy sensor and algorithm for detecting daytime sleep in poststroke individuals.

**Methods:**

Participants wore Actiwatch Spectrum and ActiGraph wGT3X-BT on their less-affected wrist, while trained observers recorded daytime sleep occurrences and activity levels (active, sedentary, and asleep) during non-therapy times. Algorithms, Actiwatch (Autoscore AMRI) and ActiGraph (Cole-Kripke, Sadeh), were compared with on-site observations and assessed using F2 scores, emphasizing sensitivity to detect daytime sleep.

**Results:**

Twenty-seven participants from an inpatient stroke rehabilitation unit contributed 173.5 hours of data. The ActiGraph Cole-Kripke algorithm (minute sleep time = 15 minutes, bedtime = 10 minutes, and wake time = 10 minutes) achieved the highest F2 score (0.59). Notably, when participants were in bed, the ActiGraph Cole-Kripke algorithm continued to outperform Sadeh and Actiwatch AMRI, with an F2 score of 0.69.

**Conclusions:**

The study demonstrates both Actiwatch and ActiGraph’s ability to detect daytime sleep, particularly during bed rest. ActiGraph (Cole-Kripke) algorithm exhibited a more balanced sleep detection profile and higher F2 scores compared to Actiwatch, offering valuable insights for optimizing daytime sleep monitoring with actigraphy in stroke patients.

**Statement of Significance**

This research addresses a gap in sleep monitoring for the poststroke population, a group prone to sleep disorders. We explore the effectiveness of actigraphy-based sensors and algorithms capturing daytime sleep (DS) in this population, which is an area lacking thorough investigation. Identifying the best-performing actigraphy-based sensor, algorithm, and parameter combinations offers immediate insights for clinical implementation. Furthermore, our study sets the stage for future research to enhance DS detection algorithms, including machine learning techniques and posture detection integration for improved accuracy. While focused on inpatient rehabilitation settings, our findings may benefit broader stroke populations, necessitating further exploration. This study contributes valuable insights into sleep monitoring for the poststroke population and underscores the importance of tailored approaches in healthcare delivery.

Sleep disorders have a devastating impact on health and well-being, with an estimated 4.1% increase in mortality and a $3461 rise in healthcare costs per affected individual [1, 2]. Stroke patients are particularly prone to higher rates of sleep disorders, such as insomnia, obstructive sleep apnea, central sleep apnea, and restless legs syndrome [3]. These conditions can disrupt sleep continuity, impede restorative sleep, and subsequently cause daytime somnolence in stroke patients [4, 5]. Increased daytime sleepiness may indicate compromised sleep quantity and quality, or the presence of underlying sleep disorders. Monitoring the presence and severity of daytime sleepiness can provide useful information for reference in clinical diagnoses of sleep disorders [6]. In a rehabilitation setting, daytime sleepiness can lead to reduced task engagement, disrupted attention, and fatigue during therapy sessions. This impairs functional performance and cognitive processes, hindering learning and memory consolidation during early stroke recovery when brain reorganization occurs [7–9]. Therefore, detection of daytime sleep (DS), particularly during acute care and inpatient rehabilitation, is crucial for timely intervention, to mitigate the adverse effects of abnormal sleep or sleep disorders on patient recovery and long-term health.

To ensure an accurate representation of DS patterns, it is essential to conduct multi-day measurements and evaluations of sleep and wake patterns. Polysomnography (PSG), the gold standard method for measuring sleep, is not practical for multi-day monitoring in the inpatient setting due to low portability, patient discomfort, and high cost [10]. Self-reporting is an easy-to-obtain method for measuring sleep and wake; however, self-reporting is subjective and often unreliable, and it is less feasible after stroke due to the prevalence of cognitive impairment and dementia (PSCID) [11, 12]. Additionally, continuously administering sleep questionnaires increases nurse burden in stroke care settings. Actigraphy offers an alternative, which records and analyzes body movement data from accelerometers to identify periods of sleep and wake [13]. Actigraphy devices, such as Actiwatch Spectrum and ActiGraph wGT3X-BT, can be worn on the wrist for weeks, offering an objective, continuous, and less-obtrusive method for long-term sleep monitoring and sleep disorder diagnosis [13, 14]. Actigraphy is an accepted method for the evaluation and management of sleep disorders in the clinic and community, and it has demonstrated consistent and objective data for measuring disorders such as insomnia and insufficient sleep syndrome in adults [13, 15]. It is yet unclear how actigraphy-based sleep–wake detection algorithms perform for individuals with stroke-capturing DS.

There are several commonly used algorithms for analyzing actigraphy data: the Actiware Philips Respironics built-in algorithm (automatic minor rest interval [AMRI]) implemented by Actiwatch, and the Cole-Kripke or Sadeh sleep scoring algorithms implemented by ActiGraph. Specifically, the Cole-Kripke algorithm was validated on healthy and sleep-disordered adults, while the Sadeh algorithm was validated on healthy adolescents and young adults, with both algorithms developed for wrist-worn sleep and wake detection [16, 17]. Poststroke physical impairments may affect the accuracy of these algorithms, since individuals with stroke often exhibit low functional capacity, including paralysis and muscle weakness on one or both sides of the body, especially during acute and subacute recovery. These impairments result in extended periods of wheelchair- or bed-bound confinement, significantly diminishing the frequency and magnitude of movements detectable by actigraphy devices and making it more difficult to distinguish between sleep and wake [18]. Furthermore, these algorithms have demonstrated low specificity in detecting wake (increased risk of type 1 errors), which is less problematic during overnight sleep but may affect DS detection performance, when sleep episodes are typically shorter and less frequent [19–21]. Thus, there is a pressing need to evaluate the efficacy of actigraphy-based devices and algorithms for DS detection.

In this study, we investigated two common wrist-worn actigraphy devices (Actiwatch and ActiGraph) and three predominantly used algorithms (Autoscore AMRI, Cole-Kripke, and Sadeh) for the detection of DS in poststroke individuals. We examined the performance of these devices and algorithms for patient subgroups based on upper body functionality and risk of EDS. The goal was to determine the best-performing device and algorithm to detect DS based on the unique circumstances and requirements of the inpatient stroke population.

## Methods

### Participants

Twenty-seven individuals with stroke (19F/8M; age 62.33 ± 3.04 years) were recruited from the inpatient unit of the Shirley Ryan AbilityLab, a rehabilitation facility in Chicago, IL (USA) between August and November 2022. Participants were required to be at least 18 years of age, have a primary stroke diagnosis, and be willing and able to provide consent to participate in the study and comply with study procedures. Patients with serious cardiac conditions, degenerative neurological pathologies, skin allergies, or severe open wounds were excluded from the study so that we could focus specifically on the effect of stroke on sleep and functional outcomes. Furthermore, patients were excluded from the study if they utilized a powered/implanted cardiac device to support heart function or were diagnosed previously with sleep disorders. Table 1 summarizes the demographics and clinical characteristics of the 27 participants.

**Table 1.** Participant Characteristics (*N* = 27)

*N* = 27	*N* (%)	Mean (SD)
Age		63.22 (3.04)
*Gender*		
Male	8 (29.63%)	
Female	19 (70.37%)	
Height		167.06 (2.01)
Weight		90.73 (7.82)
BMI		32.23 (2.54)
Length of stay		24.75 (1.73)
Days since stroke		15.40 (12.75)
*Comorbidities*		
Pulmonary	5 (18.52%)	
Diabetes	8 (29.63%)	
CHF	1 (3.70%)	
ESRD	1 (3.70%)	
MOCA		22.57 (0.86)
ISI		5.44 (1.03)
ESS		9.15 (0.99)
PSQI		7.37 (0.67)
Smoker	5 (18.52%)	
Hemorrhagic stroke	7 (25.93%)	
*Education level*		
Some high school	1 (3.70%)	
High school graduate	4 (14.81%)	
Some college	9 (33.33%)	
College graduate	5 (18.52%)	
Postgraduate	7 (25.93%)	
*Lifestyle*		
Sedentary	5 (18.52%)	
Moderately Active	12 (44.44%)	
Highly Active	9 (33.33%)	

BMI, body mass index; CHF, congestive heart failure; ESRD, end-stage renal disease; ESS, Epworth Sleepiness Scale; MOCA, Montreal Cognitive Assessment; ISI, Insomnia Severity Index; PSQI, Pittsburgh Sleep Quality Index; SD, standard deviation.

### Equipment

Participants wore the Actiwatch Spectrum (Philips, Cambridge, MA) and ActiGraph wGT3X-BT (ActiGraph, Pensacola, FL) on the dorsal wrist of their less-affected side. For medical reasons or discomfort, six participants switched their device placements: five wore the ActiGraph on their more-affected side and the Actiwatch on their less-affected side, and one participant wore the Actiwatch on their more-affected side and ActiGraph on their less-affected side. Additional analysis determined that excluding or including data from these participants did not change the study results; therefore, all participants were included in the final analysis.

### Procedures

Three trained observers obtained ground truth information about a patient’s DS or wake status using visual annotations. Other studies have successfully used trained observers to identify sleep and wake within patients [22]. The observer would annotate participants’ sleep or wake status during different times of day without disturbing or alarming the participants. During observations, participants mostly stayed in their patient room alone, except for regular check-ins from nursing staff and occasional visits from family members. Observations were excluded when the care team was providing patient assistance or conducting therapy, since there was minimal likelihood of DS during those intervals. Therapy sessions and medical check-ins occurred periodically throughout the day between 08:00 am and 05:00 pm depending on patient-specific schedules and therapy plans, with all patients completing on average two or more therapy sessions a day. All observations were during periods of rest or patient downtime between therapy or check-ins. However, the timing and occurrence of DS were patient-specific, and given the variation in patient schedules, there were no discernible trends in DS.

Two sets of data in this study were recorded at different temporal resolutions. In the first dataset (*N* = 11), participants were observed at 2-minute intervals between 09:00 am and 05:00 pm, during periods when they were not engaged in therapy sessions. We used this first set of data to conduct a power analysis and determined that we would need at least 12 participants to detect 10% changes in sensitivity, with the prevalence of DS being 5.3% obtained from our first dataset [23]. Power calculations were performed on the Actiwatch, ActiGraph with Cole-Kripke, and ActiGraph with Sadeh using the sensitivity values 0.95, 0.96, and 0.91, respectively, with an assumption of 48 (1.6 hours) observations per participant [24]. A minimum requirement of 12 participants was derived from the ActiGraph with Sadeh, thus 16 additional participants were observed to exceed the calculated requirement. In this second dataset (*N* = 16), participants were observed at 10-minute intervals between 09:00 am and 05:00 pm. Increasing observations from 2 to 10 minutes was based on the insights learned during data collection. The first includes capturing short-duration naps and dozing periods which were determined to be on average 11.2 minutes (5 instances) from the first set. The second was to remain under the 15 minimum sleep time parameter as actigraphy algorithms required a minimum sleep time of 15 minutes for both devices. Lastly, due to observations occurring for multiple patients at a time from just one observer, 10 minutes was determined to be sufficient to allow adequate and accurate observation of patient activity.

For each observation time, the observers annotated whether participants were active, sedentary, or asleep. To be categorized as asleep, participants needed to meet the following criteria: (1) sleeping with eyes closed and (2) exhibiting no voluntary movements. To avoid mistaking sedentary behavior as asleep, the first annotation of asleep required at least one full minute of observation. If the participant’s behavior during this 1-minute period matched the definition, the status would be annotated as “asleep.” Alternatively, when participants did not meet the definition but also showed no movements, they were annotated as “sedentary” (for example, watching TV without physical movements). If any limb movement was observed, those times were annotated as “active.” The observers also ensured proper actigraphy device placement during their observation times. Additionally, the location of the participants was annotated, indicating whether they were in a wheelchair or in bed. Due to observations being in between active therapy sessions or check-ins and during periods of rest, all instances of observed activity were confined to bed or in a wheelchair. All patients had access to a wheelchair for safety regardless of functional necessity, to be used for transportation to therapy sessions, bathroom and shower assistance, or in general, seating while in the room.

### Data analysis

All preprocessing and statistical analyses were performed in Python 3.9.13 with the *sklearn* library. Actiwatch data were analyzed with Autoscore AMRI sleep/wake algorithm from Actiware (version 6.2.0.39, Philips, Cambridge, MA). ActiGraph data were analyzed with two different auto-scored sleep–wake algorithms from ActiLife (version 6.13.4, Pensacola, FL): The Cole-Kripke and Sadeh algorithms [16, 17]. Each algorithm includes adjustable parameters, including: (1) the minimum duration required for a sleep period, (2) the minimum duration of inactivity to be considered in bed, and (3) the minimum duration of activity to be considered awake. Table 2 shows the values tested for each parameter in this study, which included the minimum and maximum values that ActiGraph and Actiwatch allowed. To determine the accuracy of algorithm-generated estimates of sleep and wake from these devices, we only included the device data that were available during the observation periods. Observers visually inspected the sleep–wake hypnogram to verify the alignment between their annotations and the device data.

**Table 2.** Actigraphy Algorithm Parameters

Actigraphy sensor	Algorithm	Activity threshold	Min. time of sleep	Min. time in bed	Min. time in wake
Actiwatch	Automatic minor rest interval	Low, medium high	15 or 40 minutes	N/A	N/A
ActiGraph	Cole-Kripke, Sadeh	N/A	15 or 40 minutes	5 or 10 minutes	10 minutes

Min. time of Sleep = Minimum total amount of time to determine as sleep; Min. time in bed = Minimum amount of time to be considered in bed; Min. time in wake = Minimum amount of time to be considered in wake.

Four naïve models were also created to compare actigraphy performance. Two included wake or sleep only, capable of only ever estimating just wake or just sleep, respectively. The second two models were based on the distribution of wake and sleep within the observed dataset. These models estimated sleep or wake a random percent of the time based on the ratio of sleep to wake, with random percent wake estimating wake, and random percent sleep estimating sleep.

#### Subgroup analysis.

To examine the algorithm performance for individuals with different levels of daytime sleepiness, we examined subgroups of participants using the Epworth Sleepiness Scale (ESS) [25]. The ESS asks individuals to estimate the likelihood of dozing off or falling asleep in eight different sedentary situations from no chance of dozing (0) to a high chance of dozing (3), with a final range of 0–32. A score of 10 or greater raises concern for daytime sleepiness, and a score greater than 15 indicates excessive sleepiness. Participants were assigned to the high DS group if they scored an ESS of 10 or higher; otherwise, they were assigned to the low DS group.

To examine the algorithm performance for individuals with different levels of function, we used the self-care quality indicator (SCQI) and the mobility quality indicator (MQI) [26]. The SCQI evaluates the level of disability and how much assistance is needed to perform various activities of daily living. These items are primarily related to upper limb function and movement, allowing us to directly compare whether the wrist-worn actigraphy algorithms were differentially affected by levels of upper limb function. Each item of the SCQI is scored from 1–6, ranging from total assistance (1) to total independence (6). Items included eating, oral hygiene, toilet hygiene, showering, upper body dressing, lower body dressing, and putting on footwear. We included five additional items from the MQI, including rolling left to right, sitting to lying, lying to sitting on the side of the bed, sitting to standing, and transferring from the bed to a wheelchair since they assessed patients’ upper body mobility and strength, and whether the patients were more likely to be bed- or wheelchair-bound. Overall, 12 items from these clinical indicators were used to determine participant function, with scores ranging from 12 to 72. Across the 27 participants, the mean functional score at inpatient admission was 32 (±9). Admission scores were generally lower than discharge scores because participants were earlier in the recovery process after stroke. The mean functional score at discharge was 57 (±14). We considered a moderate score at discharge to be 36 (scoring 3 on all items), and a moderate score at admission to be 27 (36 minus one standard deviation, to account for the expected lower scores at admission). Thus, participants were assigned to a low-function group if they scored below 27 at admission; otherwise, they were assigned to a high-function group. Only admission scores were considered since this would be the expected time to select an algorithm for sleep and wake monitoring, at the start of a patient’s stay.

#### Statistical metrics.

To determine the best-performing actigraphy scoring algorithm and parameters needed for each device, we calculated sensitivity, specificity, precision, and an F-score, as recommended in the literature [27]. The primary metric for evaluating each algorithm was the F2 score, which ranges from 0 (lowest performance) to 1 (highest performance) and is computed according to equation:


$$\begin{array}{l} F_{\beta }=(1+\beta^{2})\frac{precision\;\times \;sensitivity}{(\beta^{2}\;\times precision)+sensitivity\;},\;where\;\beta =2 \end{array}$$

The F2 score evaluates an algorithm based on its sensitivity and precision, with a weighted emphasis on sensitivity. F2 scores penalize false negatives more than false positives, meaning that they prioritize correctly identifying instances of sleep (minimizing false negatives), potentially with the tradeoff of mistaking more instances of wake or sedentary time as sleep. We chose the F2 score due to the infrequency of DS, making it a higher priority to catch sleep and naps during the day and thus being more forgiving of type 1 errors. In cases where F2 scores were comparable between systems, we used specificity as a secondary metric for algorithm evaluation, which measures its ability to correctly identify wake.

## Results

**Figure 1.**
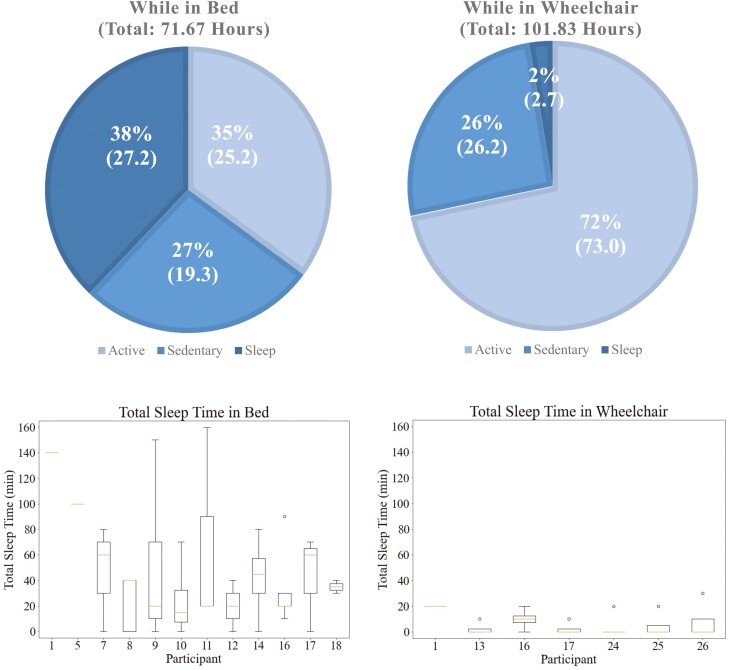
Top: Total time spent in each location, bed or wheelchair, and the associated times spent in each activity, active, sedentary, or sleep. Bottom: Total sleep by participant in bed and in wheelchair.

Data from one individual were excluded due to low adherence in wearing the devices, resulting in 26 participants for analysis. Of these, 11 were observed for 1 day at 2-minute intervals, while the remaining 15 were observed for an average of 5 days at 10-minute intervals. All 2-minute data were down-sampled to 10-minute data, which caused no significant change in the analysis. Ten participants had no DS; seven of these 10 were only observed for 1 day under the 2-minute intervals, while the other three had no DS across 4–5 days of observation. A total of 173.5 hours of data were collected: 143.7 (83%) hours were spent awake, including 98.2 (57%) hours active and 45.5 (26%) hours sedentary; 29.9 (17%) hours were spent asleep. Of the total time, 101.83 (59%) hours were spent in a wheelchair, while 71.67 (41%) hours were spent in bed. The distribution of sleep–wake activities in these locations are depicted in Figure 1. The random percent naïve models used the percent of sleep and wake as the percent to estimate wake or sleep. In the random percent wake model, wake was estimated at 83% of the time, while in the random percent sleep model, sleep was estimated at 17% of the time.

### Algorithm performance

We first compared F2 scores for detecting DS as depicted in Table 3. The highest F2 scores for both Actiwatch and ActiGraph, are displayed as well as the corresponding parameter settings. Across all data, the highest F2 score was achieved through ActiGraph with the following settings: Cole-Kripke, minimum 15 minutes of sleep time, minimum 10 minutes in bed, and minimum 10 minutes in wake (F2 score = 0.59). The overall F2 score was nearly 0.1 higher than Actiwatch (F2 score = 0.52).

**Table 3.** Statistics of Optimal Detection of Daytime Sleep

Actigraphy sensor	Condition	Algorithm	Activity threshold	Min. time of sleep (minutes)	Min. time in bed (minutes)	F2 score	Sensitivity	Specificity
Actiwatch	High DS	Auto.	Medium	15	N/A	0.49	0.54	0.70
	Low DS		High	40		0.59	0.67	0.86
	High Func.		Medium	15		0.45	0.49	0.85
	Low Func.		High	40		0.58	0.65	0.77
	Overall		Medium	15		0.52	0.57	0.80
			High	40		0.49	0.53	0.82
ActiGraph	High DS	Sadeh	N/A	15	10	0.62	0.68	0.75
	Low DS	Cole-Kripke		15	10	0.56	0.73	0.74
	High Func.	Cole-Kripke		40	10	0.60	0.73	0.77
	Low Func.	Cole-Kripke		15	10	0.60	0.72	0.67
	Overall	Cole-Kripke		15	10	0.59	0.73	0.69
		Sadeh		15	5	0.57	0.65	0.77

Min. time of Sleep = Minimum total amount of time to determine as sleep; Min. time in bed = Minimum amount of time to be considered in bed; High DS = High daytime sleep; Low DS = Low daytime sleep; High Func. = High function group, Low Func. = Low function group; Auto. = Automatically set minor intervals.

**Figure 2.**
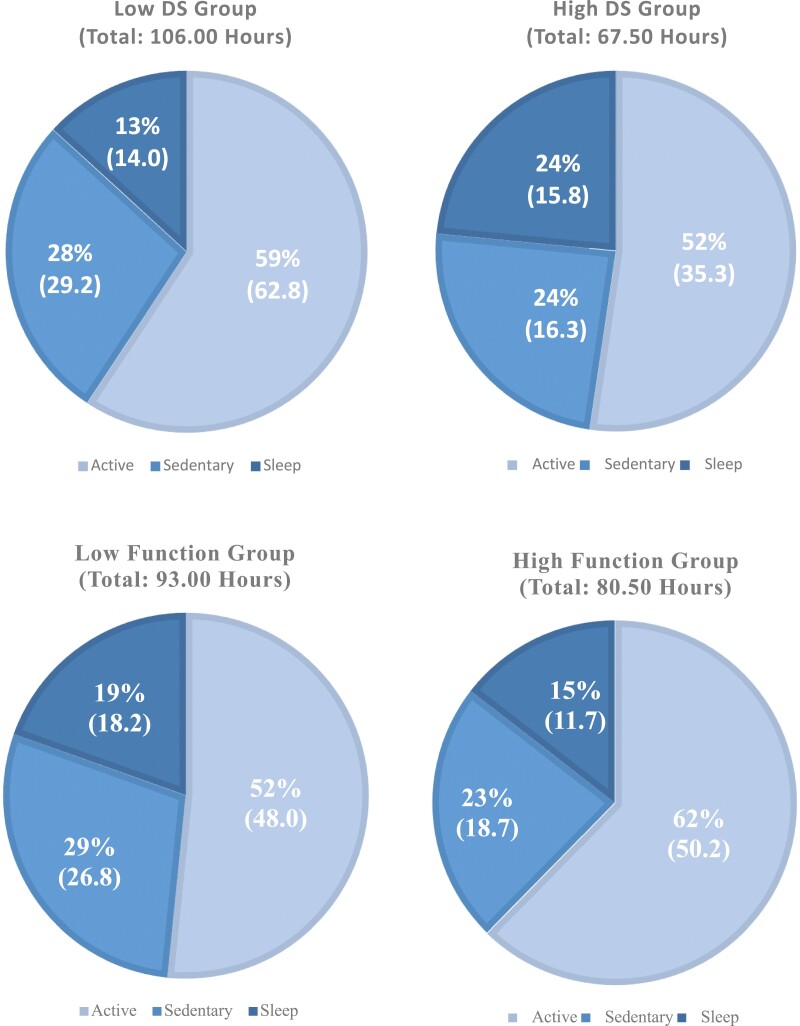
Total recorded hours by specified subgroup, displaying percent spent in each activity: active, sedentary, and sleep. Top left: low DS group, top right: high DS group, bottom left: low function group, bottom right: high function group.

In the subgroup analyses, 11 participants exhibited a high likelihood of daytime sleepiness (high DS), while 15 had a low likelihood (low DS), with the distribution of each activity by subgroup shown in Figure 2. Table 3 presents the best-performing parameters and results for both high and low DS groups. Among the high DS group, ActiGraph achieved a higher F2 score (F2 = 0.62) compared to Actiwatch (F2 = 0.49), whereas Actiwatch (F2 = 0.59) outperformed ActiGraph (F2 = 0.56) in the low DS group.

Thirteen individuals were categorized into both higher and lower function groups, with activity distributions shown in Figure 2 and corresponding results in Table 3. In the high-function group, ActiGraph attained a higher F2 score (F2 = 0.60) compared to Actiwatch (F2 = 0.45). Similarly, ActiGraph (F2 = 0.60) received a higher F2 score in the low-function group compared to Actiwatch (F2 = 0.58). Subgroups can be further described into 6 low DS low function, 9 low DS high function, 7 high DS low function, and 4 high DS high function. Due to small sample sizes, the results of each pairing were provided in Supplementary Table 1 and were not used to make final conclusions.

Across all datasets and subgroups, Actiwatch consistently exhibited higher specificity, while ActiGraph Cole-Kripke algorithm showed higher sensitivity. Notably, the Sadeh algorithm demonstrated higher specificity and lower sensitivity, resembling Actiwatch. Overall, the best-performing F2 scores and highest sensitivity were observed with ActiGraph using the Cole-Kripke algorithm.

**Figure 3.**
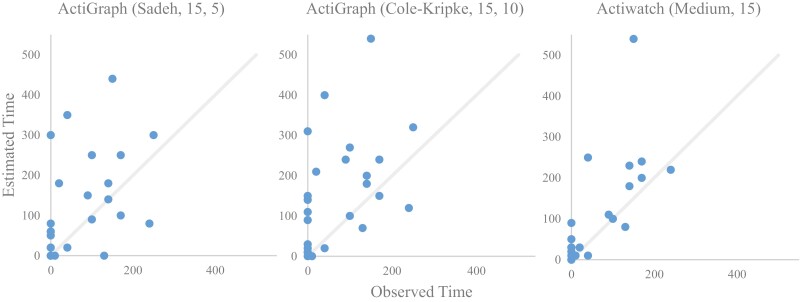
Estimated versus observed sleep time during daytime observations from the best-performing algorithm for detecting daytime sleep. Left: ActiGraph, Sadeh (minimum time in sleep = 15, minimum time in bed = 5) (F2 score = 0.57, Specificity = 0.77). Middle: ActiGraph, Cole-Kripke (minimum time in sleep = 15, minimum time in bed = 10) (F2 score = 0.59, Specificity = 0.69). Right: Actiwatch, Medium threshold (minimum time in sleep = 15) (F2 score = 0.52, Specificity = 0.80).

Figure 3 illustrates the trend of estimating DS with the best-performing algorithms across all data. Both ActiGraph Sadeh and Actiwatch algorithms tended to estimate closer to zero when observed was zero compared to the Cole-Kripke model. Actiwatch did not estimate a time greater than 100 minutes when the observed time was zero compared to the ActiGraph.

Algorithm performances were also compared to naïve algorithms. Estimating only wake results in 0 sensitivity, therefore 0 F2, so it was not used for comparison. The sleep-only naïve model, estimating only sleep, resulted in a F2 score of 0.51, with sensitivity = 1.0 and specificity = 0.0. Estimating sleep a random percent of the time based on the observed ratio of sleep to wake scored 0.16, with sensitivity = 0.16 and specificity = 0.82, while the opposite (switching sleep and wake) scored 0.48, with sensitivity = 0.83 and specificity = 0.18.

### Accuracy by location

Algorithm performance was affected by location—that is, whether the participant was in a wheelchair or in bed (Table 4, optimal algorithm only). Nine participants slept only while in bed, four slept only while in a wheelchair, and three slept both in a bed and a wheelchair (all three of which had low physical function and high risk for DS). ActiGraph outperformed Actiwatch while participants were in bed, using the same parameters of the Cole-Kripke algorithm (minimum 15 minutes of sleep time, minimum 10 minutes in bed) with an F2 score of 0.69. Distinguishing by location increased the maximum F2 score by 0.1, albeit with a slight decrease in specificity. When focusing solely on wheelchair data, Actiwatch outperformed ActiGraph; however, both performed poorly, averaging around 0.3 in F2 score. The best model for wheelchair-only data was attributed to Actiwatch with medium threshold and 40 minutes minimum sleep (F2 score = 0.34). Changes in location and the associated shift in sleep–wake proportions strongly influenced F2 and specificity. Across the full dataset, both Actiwatch and ActiGraph (Sadeh) demonstrated higher specificity scores and consequently achieved higher F2 scores when the wheelchair data predominantly recorded wakefulness.

**Table 4.** Statistics of Optimal Detection of Daytime Sleep by Location

Location	Actigraphy sensor	Condition	Algorithm	Activity threshold	Min. time of sleep (minutes)	Min. time in bed (minutes)	F2 score	Sensitivity	Specificity
Bed	Actiwatch	High DS	Auto.	Medium	15	N/A	0.54	0.53	0.63
		Low DS		High	40		0.67	0.68	0.84
		High Func.		Medium	15		0.49	0.48	0.80
		Low Func.		High	15		0.68	0.74	0.53
		Overall		Medium	15		0.58	0.58	0.72
	ActiGraph	High DS	Cole-Kripke	N/A	40	10	0.70	0.72	0.66
		Low DS			15		0.69	0.74	0.71
		High Func.			40		0.70	0.72	0.76
		Low Func.			15		0.69	0.76	0.56
		Overall			15		0.69	0.74	0.63
Wheel-chair	Actiwatch	High DS	Auto.	Medium	40	N/A	0.42	0.56	0.91
		Low DS		Low	15		0.33	0.43	0.96
		High Func.		Low	15		0.44	0.60	0.96
		Low Func.		Medium	40		0.36	0.45	0.93
		Overall		Medium	40		0.34	0.50	0.93
	ActiGraph	High DS	Sadeh	N/A	40	10	0.32	0.56	0.81
		Low DS					0.24	0.57	0.87
		High Func.					0.30	0.80	0.86
		Low Func.					0.26	0.45	0.84
		Overall					0.28	0.56	0.85

Min. time of Sleep = Minimum total amount of time to determine as sleep; Min. time in bed = Minimum amount of time to be considered in bed; High DS = High daytime sleep; Low DS = Low daytime sleep; High Func. = High function group, Low Func. = Low function group; Auto. = Automatically set minor intervals.

A similar trend emerged across subgroups, with higher F2 scores observed in bed compared to a wheelchair. For the high DS group, approximately 52.6% of the time was spent in a wheelchair, increasing to 62.6% in the low DS group. The high-functioning group spent 62.9% of the time in a wheelchair, while the low-functioning group spent only 55%. Looking at the combination of subgroups, 50.7% (of 48.7 hours) low DS low function, 72.6% (of 57.3 hours) low DS high function, 59.8% (of 44.3 hours) high DS low function, and 38.8% (of 23.2 hours) high DS high function of time was spent in a wheelchair.

In summary, the participant’s location significantly impacted accuracy, with actigraphy algorithms demonstrating higher predictive power when the patient was in bed.

## Discussion

In this study, we tested three different algorithms from two commonly used actigraphy devices and compared their ability to detect DS in inpatients with stroke. This study provides evidence of accuracies for DS using different algorithms and parameter settings. We found that ActiGraph (best F2 = 0.59) generally achieved better performance than Actiwatch (best F2 = 0.52). Actiwatch, however, tended to have higher specificity (specificity of best settings = 0.80) than ActiGraph. For ActiGraph, the Sadeh algorithm demonstrated greater specificity (specificity of best settings = 0.77) than the Cole-Kripke algorithm (specificity of best settings = 0.69), but at the cost of a lower F2 and sensitivity (F2 = 0.57 vs. F2 = 0.59, sensitivity = 0.65 vs. sensitivity = 0.73, respectively) which were considered more important in capturing DS given the lower prevalence of sleep during the day. Both Actiwatch and ActiGraph outperformed the naïve models.

This study suggests a reversal of the typical performance characteristics associated with actigraphy devices compared to previous research in healthy adult populations. In this study, ActiGraph exhibited high sensitivity but low specificity, in contrast to the low sensitivity and higher specificity reported in healthy adults. Prior studies have often favored Actiwatch devices as superior for assessing both daytime and nighttime sleep [14, 28]. Furthermore, a notable shift in sensitivity and specificity metrics between Sadeh and Cole-Kripke algorithms was evident in the stroke population when compared to healthy adults [29]. We propose that this reversed relationship may be influenced by various factors, including the specific make and model of ActiGraph or Actiwatch devices used, the unique study environment of a rehabilitation hospital, and importantly, the intrinsic characteristics of the stroke population itself. It remains unclear whether the findings about the best-performing algorithms for detecting DS can be generalized to nighttime sleep in a stroke population.

Comparing subgroups, given the slight increases in F2 scores, and the distribution of activity, there is promise in future exploration of subgroups as the high DS group and low function group slept more and spent more time in bed than their counterparts. The cutoffs using ESS and QI are easily administered and help inform future models based on the higher chance of daytime sleepiness and functional ability. Perhaps a better subgroup would be to focus more on the patient’s location.

Between in bed or in a wheelchair, the proportion of sleep observations compared to wake observations was drastically different with only around 3 (2% of 102 hours) hours of sleep occurring in the wheelchair, versus 27 (38% of 72) hours of sleep occurring in bed across all participants. The instances of sleep in a wheelchair were also much shorter in duration, with an average near the observation time of ten minutes. Sleep occurred more frequently and lasted for longer durations, averaging around 40 minutes, while in bed. Objectively, the F2 scores increase for both ActiGraph and Actiwatch when looking at bed-only data, with ActiGraph scoring an F2 of 0.69, a 0.1 improvement when considering both bed and wheelchair. Sleep detection while in a wheelchair remained low, with the best F2 score with Actiwatch at 0.34, which given the high specificity of 0.93 and disproportionate wake-to-sleep data, supports Actiwatch at better estimating wake periods. While only looking at in-bed data, the naïve all sleep estimation received an F2 = 0.75, sensitivity = 1, and specificity = 0 which slightly outperformed the best ActiGraph model; however, it is incapable of estimating wake, making it detrimental to sleep-only estimation. All sleep when looking at the wheelchair only scored F2 = 0.12, sensitivity = 1, and specificity = 0.03, which was outperformed by both ActiGraph and Actiwatch.

Improvements to this study and the detection of DS in stroke would include healthy controls for comparison or data-driven modeling. Healthy controls would allow for results to be contrasted with patients with stroke which may suggest future subgroup considerations and extrapolation beyond acute stroke contexts. Through data-driven modeling, it would be possible to deploy posture detection to determine if a patient is in bed or in a wheelchair to improve the DS detection with actigraphy. Future work can look into the use of the raw accelerometer signal from ActiGraph to detect posture and only check for sleep while in bed or in low-motion activity. Data-driven modeling would also allow for higher resolution estimations outside of just sleep and wake and could inform on activity such as sedentary or short nap duration and DS-staging. These approaches would allow for better continuous monitoring of stroke patients’ sleep patterns during the day which have not been considered as often as nighttime sleep alone. This study did not use PSG, the gold standard for sleep detection, which would be required for future projects.

Several limitations exist in our comparison of Actiwatch and ActiGraph devices. The study’s sample size is small, consisting of only 26 participants, resulting in 173.5 hours of monitoring that predominantly captured wakefulness. Additionally, the recorded sleep/wake data does not resemble that of a typical day, as it was only observed during patient downtime outside of care and therapy sessions. Nonetheless, a power analysis based on the prevalence rates ensured sufficient sample hours, providing evidence of the overall accuracy of sensor-based DS detection. Although this study cannot draw conclusions about DS trends and or time effects, the observations effectively captured the periods when DS is most likely among stroke patients. Both Actiwatch and ActiGraph devices were challenged to detect short-duration naps, as their algorithms typically require a minimum of 15 minutes to identify a sleep event. Many short naps occurring outside of bed settings may fall below this minimum threshold, resulting in an underestimation of sleep duration. Adjusting the minimum threshold to 40 minutes often enhanced specificity, favoring the identification of data as wake by necessitating four consecutive observations of sleep before classifying an event as sleep. Overall this study assessed the performance of two commercial actigraphy sensors, and three separate algorithms at detecting DS in patients with stroke. Insights and findings from this research inform future directions and decisions regarding DS detection during acute rehabilitation and potentially beyond. The need to quantify DS and monitor overall sleep structure, provides important information about overall healing of brain systems, and can add important information for medical management poststroke.

## Supplementary Material

zpae057_suppl_Supplementary_Tables

## References

[1] Huyett P , Siegel N, Bhattacharyya N. Prevalence of sleep disorders and association with mortality: results from the NHANES 2009–2010. Laryngoscope. 2021;131(3):686–689. doi: 10.1002/lary.28900 32681735

[2] Hui S kuen A , Grandner MA. Trouble sleeping associated with lower work performance and greater health care costs: longitudinal data from Kansas state employee wellness program. J Occup Environ Med. 2015;57(10):1031. doi: 10.1097/JOM.0000000000000534 26461857 PMC4610176

[3] Cai H , Wang XP, Yang GY. Sleep disorders in stroke: an update on management. Aging Dis. 2021;12(2):570–585. doi: 10.14336/AD.2020.0707 33815883 PMC7990374

[4] Bogan RK. Effects of restless legs syndrome (RLS) on sleep. Neuropsychiatr Dis Treat. 2006;2(4):513–519. doi: 10.2147/nedt.2006.2.4.513. https://www.ncbi.nlm.nih.gov/pmc/articles/PMC2671944/. Accessed June 12, 2024. 19412499 PMC2671944

[5] Lal C , Weaver TE, Bae CJ, Strohl KP. Excessive daytime sleepiness in obstructive sleep apnea. mechanisms and clinical management. Ann Am Thorac Soc. 2021;18(5):757–768. doi: 10.1513/AnnalsATS.202006-696FR 33621163 PMC8086534

[6] Slater G , Steier J. Excessive daytime sleepiness in sleep disorders. J Thorac Dis. 2012;4(6):608–616. doi: 10.3978/j.issn.2072-1439.2012.10.07 23205286 PMC3506799

[7] Ding Q , Whittemore R, Redeker N. Excessive daytime sleepiness in stroke survivors. Biol Res Nurs. 2016;18(4):420–431. doi: 10.1177/1099800415625285 26792913 PMC6344831

[8] Hermann DM , Siccoli M, Brugger P, et al. Evolution of neurological, neuropsychological and sleep-wake disturbances after paramedian thalamic stroke. Stroke. 2008;39(1):62–68. doi: 10.1161/STROKEAHA.107.494955 18048862

[9] Sterr A , Herron K, Dijk DJ, Ellis J. Time to wake-up: sleep problems and daytime sleepiness in long-term stroke survivors. Brain Inj. 2008;22(7-8):575–579. doi: 10.1080/02699050802189727 18568710

[10] Cooksey JA , Balachandran JS. Portable monitoring for the diagnosis of OSA. Chest. 2016;149(4):1074–1081. doi: 10.1378/chest.15-1076 26539918

[11] Kawada T. Agreement rates for sleep/wake judgments obtained via accelerometer and sleep diary: a comparison. Behav Res Methods. 2008;40(4):1026–1029. doi: 10.3758/BRM.40.4.1026 19001393

[12] Rost NS , Brodtmann A, Pase MP, et al. Post-stroke cognitive impairment and dementia. Circ Res. 2022;130(8):1252–1271. doi: 10.1161/CIRCRESAHA.122.319951 35420911

[13] Smith MT , McCrae CS, Cheung J, et al. Use of Actigraphy for the evaluation of sleep disorders and circadian rhythm sleep-wake disorders: an American Academy of sleep medicine clinical practice guideline. J. Clin. Sleep Med. 2018;14(7):1231–1237. doi: 10.5664/jcsm.7230 29991437 PMC6040807

[14] Gao C , Li P, Morris CJ, et al. Actigraphy-based sleep detection: validation with polysomnography and comparison of performance for nighttime and daytime sleep during simulated shift work. Nat Sci Sleep. 2022;14:1801–1816. doi: 10.2147/NSS.S373107 36275180 PMC9581540

[15] Morgenthaler T , Alessi C, Friedman L, et al.; Standards of Practice Committee. Practice parameters for the use of actigraphy in the assessment of sleep and sleep disorders: an update for 2007. Sleep. 2007;30(4):519–529. doi: 10.1093/sleep/30.4.519 17520797

[16] Cole RJ , Kripke DF, Gruen W, Mullaney DJ, Gillin JC. Automatic sleep/wake identification from wrist activity. Sleep. 1992;15(5):461–469. doi: 10.1093/sleep/15.5.461 1455130

[17] Sadeh A , Sharkey KM, Carskadon MA. Activity-based sleep-wake identification: an empirical test of methodological issues. Sleep. 1994;17(3):201–207. doi: 10.1093/sleep/17.3.201 7939118

[18] Conley S , Knies A, Batten J, et al. Agreement between actigraphic and polysomnographic measures of sleep in adults with and without chronic conditions: a systematic review and meta-analysis. Sleep Med Rev. 2019;46:151–160. doi: 10.1016/j.smrv.2019.05.001 31154154 PMC6594867

[19] Marino M , Li Y, Rueschman MN, et al. Measuring sleep: accuracy, sensitivity, and specificity of wrist actigraphy compared to polysomnography. Sleep. 2013;36(11):1747–1755. doi: 10.5665/sleep.3142 24179309 PMC3792393

[20] de Souza L , Benedito-Silva AA, Pires MLN, Poyares D, Tufik S, Calil HM. Further validation of actigraphy for sleep studies. Sleep. 2003;26(1):81–85. doi: 10.1093/sleep/26.1.81 12627737

[21] Chakar B , Senny F, Poirrier AL, Cambron L, Fanielle J, Poirrier R. Validation of midsagittal jaw movements to measure sleep in healthy adults by comparison with actigraphy and polysomnography. Sleep Sci. 2017;10(3):122–127. doi: 10.5935/1984-0063.20170021 29410741 PMC5699855

[22] Carroll JS , Bliwise DL, Dement WC. A method for checking interobserver reliability in observational sleep studies. Sleep. 1989;12(4):363–367. doi: 10.1093/sleep/12.4.363 2762690

[23] Hajian-Tilaki K. Sample size estimation in diagnostic test studies of biomedical informatics. J Biomed Inform. 2014;48:193–204. doi: 10.1016/j.jbi.2014.02.013 24582925

[24] Quante M , Kaplan ER, Cailler M, et al. Actigraphy-based sleep estimation in adolescents and adults: a comparison with polysomnography using two scoring algorithms. Nat Sci Sleep. 2018;10:13–20. doi: 10.2147/NSS.S151085 29403321 PMC5779275

[25] Johns MWA. new method for measuring daytime sleepiness: the Epworth sleepiness scale. Sleep. 1991;14(6):540–545. doi: 10.1093/sleep/14.6.540 1798888

[26] Deutsch A , Palmer L, Vaughan M, et al. Inpatient rehabilitation facility change in self-care and change in mobility quality measures: development and reliability and validity testing. Arch Phys Med Rehabil. 2022;103(6):1105–1112. doi: 10.1016/j.apmr.2021.12.031 35143748

[27] Chinchor N. MUC-4 evaluation metrics. In: Proceedings of the 4th Conference on Message Understanding. MUC4’92. Kerrville, Texas, USA: Association for Computational Linguistics; 1992:22–29. doi: 10.3115/1072064.1072067

[28] Kanady JC , Drummond SPA, Mednick SC. Actigraphic assessment of a polysomnographic-recorded nap: a validation study. J Sleep Res. 2011;20(1 Pt 2):214–222. doi: 10.1111/j.1365-2869.2010.00858.x 20626612

[29] Lee PH , Suen LKP. The convergent validity of Actiwatch 2 and ActiGraph Link accelerometers in measuring total sleeping period, wake after sleep onset, and sleep efficiency in free-living condition. Sleep Breath. 2017;21(1):209–215. doi: 10.1007/s11325-016-1406-0 27614441

