# Supplementary material for: Assessing actigraphy performance for daytime sleep detection following stroke: insights from inpatient monitoring in a rehabilitation hospital
Source: Sleep Adv. 2024 Jul 31;5(1):zpae057. doi: 10.1093/sleepadvances/zpae057 (PMC11331150; doi:10.1093/sleepadvances/zpae057)
Supplement: zpae057_suppl_Supplementary_Tables [file zpae057_suppl_supplementary_tables.docx]

| **N=27** | **N (%)** | **Mean (SD)** |
| --- | --- | --- |
| Age |  | 63.22 (3.04) |
| Gender |  |  |
| Male | 8 (29.63%) |  |
| Female | 19 (70.37%) |  |
| Height |  | 167.06 (2.01) |
| Weight |  | 90.73 (7.82) |
| BMI |  | 32.23 (2.54) |
| Length of Stay |  | 24.75 (1.73) |
| Days Since Stroke |  | 15.40 (12.75) |
| Comorbidities |  |  |
| Pulmonary | 5 (18.52%) |  |
| Diabetes | 8 (29.63%) |  |
| CHF | 1 (3.70%) |  |
| ESRD | 1 (3.70%) |  |
| MOCA |  | 22.57 (0.86) |
| ISI |  | 5.44 (1.03) |
| ESS |  | 9.15 (0.99) |
| PSQI |  | 7.37 (0.67) |
| Smoker | 5 (18.52%) |  |
| Hemorrhagic Stroke | 7 (25.93%) |  |
| Education Level |  |  |
| Some high school | 1 (3.70%) |  |
| High school graduate | 4 (14.81%) |  |
| Some college | 9 (33.33%) |  |
| College graduate | 5 (18.52%) |  |
| Post graduate | 7 (25.93%) |  |
| Lifestyle |  |  |
| Sedentary | 5 (18.52%) |  |
| Moderately Active | 12 (44.44%) |  |
| Highly Active | 9 (33.33%) |  |
| BMI- Body Mass Index, CHF- Congestive Heart Failure,  ESRD-End Stage Renal Disease, ESS- Epworth Sleepiness Scale  MOCA- Montreal Cognitive Assessment, ISI- Insomnia Severity Index,  PQSI- Pittsburgh Quality Sleep Index, SD- Standard Deviation | | |

***Table 1: Participant Characteristics (N=27)*.**

| ***Table 2: Actigraphy Algorithm Parameters*.** | | | | | |
| --- | --- | --- | --- | --- | --- |
| **Actigraphy Sensor** | **Algorithm** | **Activity Threshold** | **Min. duration of Sleep** | **Min. duration of inactivity** | **Min. duration of activity** |
| ActiWatch | Automatic minor rest interval | Low, Medium High | 15 or 40 minutes | N/A | N/A |
| ActiGraph | Cole Kripke, Sadeh | N/A | 15 or 40 minutes | 5 or 10 minutes | 10 minutes |
| Min. time of Sleep = Minimum total amount of time to determine as sleep; Min. time in bed = Minimum amount of time to be considered in bed; Min. time in wake = Minimum amount of time to be considered in Wake | | | | | |

***Table 3: Statistics of optimal detection of daytime sleep.***

| Actigraphy Sensor | Condition | Algorithm | Activity Threshold | Min. time of Sleep (minutes) | Min. time in bed (minutes) | F2 Score | Sensitivity | Specificity |
| --- | --- | --- | --- | --- | --- | --- | --- | --- |
| ActiWatch | High DS | Auto. | Medium | 15 | N/A | 0.49 | 0.54 | 0.70 |
|  | Low DS |  | High | 40 |  | 0.59 | 0.67 | 0.86 |
|  | High Func. |  | Medium | 15 |  | 0.45 | 0.49 | 0.85 |
|  | Low Func. |  | High | 40 |  | 0.58 | 0.65 | 0.77 |
|  | Overall |  | Medium | 15 |  | 0.52 | 0.57 | 0.80 |
|  |  |  | High | 40 |  | 0.49 | 0.53 | 0.82 |
| ActiGraph | High DS | Sadeh | N/A | 15 | 10 | 0.62 | 0.68 | 0.75 |
|  | Low DS | Cole Kripke |  | 15 | 10 | 0.56 | 0.73 | 0.74 |
|  | High Func. | Cole Kripke |  | 40 | 10 | 0.60 | 0.73 | 0.77 |
|  | Low Func. | Cole Kripke |  | 15 | 10 | 0.60 | 0.72 | 0.67 |
|  | Overall | Cole Kripke |  | 15 | 10 | 0.59 | 0.73 | 0.69 |
|  |  | Sadeh |  | 15 | 5 | 0.57 | 0.65 | 0.77 |
| Min. time of Sleep = Minimum total amount of time to determine as sleep; Min. time in bed = Minimum amount of time to be considered in bed; High DS = High daytime sleep; Low DS = Low daytime sleep; High Func. = High function group, Low Func. = Low function group; Auto. = Automatically set minor interval | | | | | | | | |

| Location | Actigraphy Sensor | Condition | Algorithm | Activity Threshold | Min. time of Sleep (minutes) | Min. time in bed (minutes) | F2 Score | Sensitivity | Specificity |  |
| --- | --- | --- | --- | --- | --- | --- | --- | --- | --- | --- |
| Bed | ActiWatch | High DS | Auto. | Medium | 15 | N/A | 0.54 | 0.53 | 0.63 |  |
|  |  | Low DS |  | High | 40 |  | 0.67 | 0.68 | 0.84 |  |
|  |  | High Func. |  | Medium | 15 |  | 0.49 | 0.48 | 0.80 |  |
|  |  | Low Func. |  | High | 15 |  | 0.68 | 0.74 | 0.53 |  |
|  |  | Overall |  | Medium | 15 |  | 0.58 | 0.58 | 0.72 |  |
|  | ActiGraph | High DS | Cole Kripke | N/A | 40 | 10 | 0.70 | 0.72 | 0.66 |  |
|  |  | Low DS |  |  | 15 |  | 0.69 | 0.74 | 0.71 |  |
|  |  | High Func. |  |  | 40 |  | 0.70 | 0.72 | 0.76 |  |
|  |  | Low Func. |  |  | 15 |  | 0.69 | 0.76 | 0.56 |  |
|  |  | Overall |  |  | 15 |  | 0.69 | 0.74 | 0.63 |  |
| Wheel-chair | ActiWatch | High DS | Auto. | Medium | 40 | N/A | 0.42 | 0.56 | 0.91 |  |
|  |  | Low DS |  | Low | 15 |  | 0.33 | 0.43 | 0.96 |  |
|  |  | High Func. |  | Low | 15 |  | 0.44 | 0.60 | 0.96 |  |
|  |  | Low Func. |  | Medium | 40 |  | 0.36 | 0.45 | 0.93 |  |
|  |  | Overall |  | Medium | 40 |  | 0.34 | 0.50 | 0.93 |  |
|  | ActiGraph | High DS | Sadeh | N/A | 40 | 10 | 0.32 | 0.56 | 0.81 |  |
|  |  | Low DS |  |  |  |  | 0.24 | 0.57 | 0.87 |  |
|  |  | High Func. |  |  |  |  | 0.30 | 0.80 | 0.86 |  |
|  |  | Low Func. |  |  |  |  | 0.26 | 0.45 | 0.84 |  |
|  |  | Overall |  |  |  |  | 0.28 | 0.56 | 0.85 |  |
| Min. time of Sleep = Minimum total amount of time to determine as sleep; Min. time in bed = Minimum amount of time to be considered in bed; High DS = High daytime sleep; Low DS = Low daytime sleep; High Func. = High function group, Low Func. = Low function group; Auto. = Automatically set minor interval | | | | | | | | | | |

***Table 4: Statistics of optimal detection of daytime sleep by location.***
